# Differential infectivity of gametocytes after artemisinin-based combination therapy of uncomplicated falciparum malaria

**DOI:** 10.4102/ajlm.v7i2.784

**Published:** 2018-12-06

**Authors:** Dinkorma T. Ouologuem, Cheick O. Kone, Bakary Fofana, Bakary Sidibe, Amadou H. Togo, Demba Dembele, Sekou Toure, Sekou Koumare, Ousmane Toure, Issaka Sagara, Abdoulaye Toure, Adama Dao, Ogobara K. Doumbo, Abdoulaye A. Djimde

**Affiliations:** 1 Malaria Research and Training Center, Department of Epidemiology of Parasitic Diseases, University of Science, Techniques and Technologies of Bamako, Bamako, Mali

## Abstract

**Background:**

Most malaria-endemic countries use artemisinin-based combination therapy (ACT) as their first-line treatment. ACTs are known to be highly effective on asexual stages of the malaria parasite. Malaria transmission and the spread of resistant parasites depend on the infectivity of gametocytes. The effect of the current ACT regimens on gametocyte infectivity is unclear.

**Objectives:**

This study aimed to determine the infectivity of gametocytes to *Anopheles gambiae* following ACT treatment in the field.

**Methods:**

During a randomised controlled trial in Bougoula-Hameau, Mali, conducted from July 2005 to July 2007, volunteers with uncomplicated malaria were randomised to receive artemether-lumefantrine, artesunate-amodiaquine, or artesunate-sulfadoxine/pyrimethamine. Volunteers were followed for 28 days, and gametocyte carriage was assessed. Direct skin feeding assays were performed on gametocyte carriers before and after ACT administration.

**Results:**

Following artemether-lumefantrine treatment, gametocyte carriage decreased steadily from Day 0 to Day 21 post-treatment initiation. In contrast, for the artesunate-amodiaquine and artesunate-sulfadoxine/pyrimethamine arms, gametocyte carriage increased on Day 3 and remained constant until Day 7 before decreasing afterward. Mosquito feeding assays showed that artemether-lumefantrine and artesunate-amodiaquine significantly increased gametocyte infectivity to *Anopheles gambiae sensu lato* (s.l.) (*p* < 10^−4^), whereas artesunate-sulfadoxine/pyrimethamine decreased gametocyte infectivity in this setting (*p* = 0.03).

**Conclusion:**

Different ACT regimens could lead to gametocyte populations with different capacity to infect the *Anopheles* vector. Frequent assessment of the effect of antimalarials on gametocytogenesis and gametocyte infectivity may be required for the full assessment of treatment efficacy, the potential for spread of drug resistance and malaria transmission in the field.

## Background

Malaria is still a major public health problem in numerous parts of the world. Malaria still affects 216 000 million individuals each year with 445 000 deaths worldwide.^1^ The global agenda for malaria elimination and eradication may never succeed without a thorough understanding of gametocyte biology and the true effect of the various interventions on malaria transmission. Gametocyte development and viability are essential for the perpetuation of *Plasmodium* life cycle by enabling both transmission from the human host to the mosquito vector^2,3^ and the spread of resistant parasites.

*Plasmodium* gametocyte development within the human host is a tedious process involving the differentiation from asexual to sexual forms to accommodate metabolic requirements, environmental changes and sexual reproduction.^4,5^ *Plasmodium* gametocytes are conventionally classified into five distinct stages (stages I–V) but only the immature stage I gametocytes and the mature stage V gametocytes are detectable in the peripheral blood of a malaria-infected patient.^6,7^ The other stages (stage II, III, IV) are sequestered in the bone marrow and possibly other internal organs.^6,8,9^ Gametocytes do not cause any symptoms in the infected human host, but the presence of competent circulating gametocytes and their duration in the bloodstream, which varies from 3 to 4 weeks,^10^ are directly responsible for malaria parasite transmission to the *Anopheles* vector.^11^ However, gametocytogenesis and gametocyte transmission to the mosquito vector constitute a population bottleneck in the *Plasmodium* life cycle as only a minute number of parasites enter the mosquito bloodmeal and gut.^12^ Monitoring the density and infectiousness of circulating gametocytes is necessary for a better assessment of malaria transmission in endemic areas.

Gametocyte development within the human host is influenced by various factors, including host and parasite genetic factors, immune response, mosquitoes’ gut microbiota and the exposure to antimalarial drugs.^13,14,15^ Several clinical and *in vitro* studies reveal that most antimalarial drugs currently in use can promote or impair gametocytogenesis and to some extent affect sexual reproduction within the mosquito vector.^16^ The 8-aminoquinoline primaquine is presently the only clinically used antimalarial drug displaying potent activity against all *Plasmodium* species and gametocyte stages,^16^ but its side effects on glucose-6-phosphate dehydrogenase-deficient individuals hinder its use in large-scale elimination strategies.^17,18^ The 4-aminoquinoline chloroquine was shown to increase the production of fully competent gametocytes both *in vitro* and *in vivo*.^19,20^ In contrast atovaquone, artemisinin and the antifolates (sulfadoxine and pyrimethamine) have been shown to impair gametocyte development and infectivity.^20,21,22,23,24^ The gametocyte developmental stages affected by antimalarial drugs is poorly understood. The antimalarial treatment represents a stress factor that triggers differentiation of the asexual form into the gametocytes.^25,26^ This process may be more prevalent with drug-resistant parasites compared to sensitive ones.^27^ Hence, the selective pressure exerted by the antimalarial drugs on the parasite may contribute to the spread of resistant parasites through the development and transmission of drug-resistant gametocytes.^28^ Therefore it becomes essential to assess the emergence of resistant strains and the impact of treatment on gametocytogenesis and gametocyte infectivity.

Artemisinin-based combination therapies (ACT) are recommended in most malaria-endemic countries^29^ with the expected benefit to reduce gametocyte carriage.^30^ Indeed, the fast killing action of artemisinin and derivatives on asexual parasites results in the decrease of circulating stage V gametocytes.^30^ Artesunate has been reported to reduce post-treatment transmission of gametocyte to *Anopheles* mosquitoes but does not abolish gametocyte infectivity entirely.^31^

With the increased interest in malaria elimination, understanding the impact of ACT regimens and other antimalarial drugs with different pharmacodynamic properties on gametocyte development and transmission becomes a key issue. Here, we report an *in vivo* assessment of the impact of artemether-lumefantrine, artesunate-amodiaquine and artesunate-sulfadoxine-pyrimethamine on circulating gametocytes density and their infectivity.

## Methods

### Ethical considerations

The protocol (NCT00452907 on ClinicalTrials.gov) was reviewed and approved by the ethical committee of the Faculty of Medicine, Pharmacy, and Dentistry, University of Bamako (No 05-20 dated 22 June 2005). Each participant (or legal guardian for minors) gave fully informed written consent before enrolment.

### Study sites

This study was conducted in Bougoula-Hameau, a peri-urban village of approximately 7000 people located near the city of Sikasso in southern Mali. *Plasmodium falciparum* is hyperendemic with seasonal peaks in this village. Parasitemia prevalence rates range from 40% to 50% during the dry season (January–April) and 70% to 85% during the rainy season (May–December).^32^ Approximately 10% – 20% of the local population are gametocyte positive with an average gametocyte density of 23 gametocytes/µl.^33^ The main malaria vectors in the Sikasso region are *Anopheles gambiae* and *Anopheles funestus* with a sporozoite rate of 6.4% at the end of the rainy season and an entomological inoculation rate (EIR) of 0.032 infected bites per person per night.^34^

### Study design, volunteer follow-up and sample collection

This is a sub-study of a previously published randomised controlled clinical trial, which was conducted from July 2005 to July 2007 to compare the efficacy of three ACT regimens: artemether + lumefantrine (Coartem^®^, Novartis, Basel, Switzerland), artesunate + amodiaquine (Arsucam^®^, Sanofi-Aventis, Paris, France), and artesunate (Sanofi-Aventis, Paris, France) + sulfadoxine-pyrimethamine (Fansidar^®^, Roche, Burlington, North Carolina, United States).^35^ Briefly, patients aged 6 months and above were enrolled in the clinical study if they satisfied the following inclusion criteria: weighed ≥ 35 kg, resided in the study village, were able to receive oral treatment, had an axillary temperature ≥ 37.5°C, and had *Plasmodium sp*. infection with a parasite density between 2,000 and 200,000 asexual forms per microlitre of blood. As described previously by Sagara et al.,^35^ 780 volunteers were enrolled and 260 were randomly assigned to one of the three treatment arms and drug efficacy was assessed according to the World Health Organization 2003 protocols.^36^ Briefly, blood samples were collected on Days 1, 2, 3, 7, 14, 21, 28 and any day of recurrent illness. Smears were made, Giemsa-stained and read on site for asexual parasites and gametocytes quantification. Parasite count was performed against 300 leucocytes and gametocyte count was performed against 1000 leucocytes.

To evaluate gametocyte carriage following ACT administration, all participants who were gametocyte carriers at Day 0, 1, 2, 3, 7, 14, 21 and 28 were selected.

### Direct skin feeding procedures

Gametocyte infectivity was defined as the presence of oocyst in the midgut of the *Anopheles* mosquito 8 days after a gametocyte containing blood meal. The infectivity of circulating gametocytes following treatment was evaluated by direct mosquito feeding experiments performed on gametocyte carriers before and after oral ACT administration on Day 0 and Days 3, 7, 14, 21 or 28. The test group was recruited among patients randomised in the main study,^35^ while the control group was recruited among patients presenting with malaria symptoms, who were gametocyte positive by microscopy but were not included in the main study. Gametocytes carriers from the control group did not receive any antimalarial drugs at the time of infectivity assessment. To avoid repeated exposure to direct skin feeding, each volunteer underwent direct skin feeds only once during the entire study. In addition, the infectivity experiments were only performed on those days when adequate numbers of 3–5 days old mosquitoes were available to the team.

Wild female *Anopheles gambiae s.l.* collected from the same study site were allowed to lay eggs. Mosquitoes were kept in semi-natural conditions in field insectaries set up in the village. For each feeding experiment, 3–5 days old offspring (F1) reared in the insectaries were used. Up to 60 F1 female mosquitoes were starved for about 12 h and held in 2 small screened-cups containing 30 mosquitoes each. They were then allowed to blood-feed for 5–10 min on the leg of a 6–18 years old consenting volunteer.

After feeding, unfed mosquitoes were removed the same day, and only fed mosquitoes were kept in the insectaries as described above. 8 days post feeding, a group of at least 15 mosquitoes per carrier were dissected, their midguts pulled out in 0.5% mercurochrome for oocyst detection and quantification. Oocyst numbers, abdomen stages, date and other observations were recorded on data sheets. Oocysts were counted under light microscopes 10 times for each mosquito.

### Statistical analysis

For the analysis of the direct skin feeding experiment, feeding experiments for Days 3–28 were lumped together in each treatment arm. To calculate the oocyst prevalence, for each treatment arm, the number of positive mosquitoes was divided by the total number of mosquitoes dissected for that treatment arm). The oocyst positivity for each ACT regimen was compared to that of the control group using the chi-square test. All data were analysed and reported using Stata software version 14.0 (StataCorp. 2015, College Station, Texas, United States).

## Results

### Evolution of gametocyte carriage and gametocyte density following artemisinin-based combination therapy treatment

**FIGURE 1:**
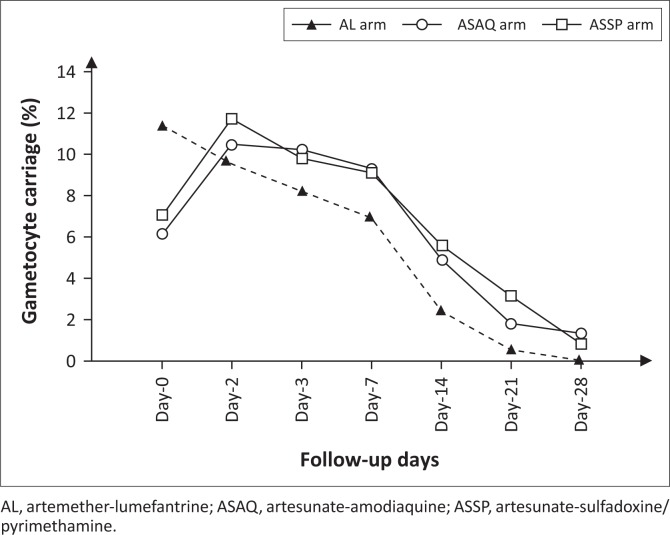
Gametocytemia carriage evolution by treatment arm.

A total of 129 volunteers were gametocyte carriers in the artemether-lumefantrine arm, 123 in the artesunate-amodiaquine arm and 124 in the artesunate-sulfadoxine-pyrimethamine arm. Following treatment initiation, gametocyte carriage significantly increased in the artesunate-amodiaquine and artesunate-sulfadoxine-pyrimethamine arms (Figure 1). From Day 0 to Day 3, gametocyte carriage rose from 6.1% to 10.2% in the artesunate-amodiaquine arm (*p* = 0.005) and from 7.0% to 9.8% in the artesunate-sulfadoxine-pyrimethamine arm (*p* = 0.02). Overall, the prevalence of gametocyte carriage in the artemether-lumefantrine arm steadily decreased from Day 0 to Day 28, while for the artesunate-amodiaquine and artesunate-sulfadoxine-pyrimethamine arms the prevalence of gametocyte carriage increased from Day 0 to Day 7 before declining (Figure 1).

### Infectivity of gametocytes before and after treatment with artemisinin-based combination therapy regimens

Overall 21 consenting volunteers in the control arm, 8 in the artemether-lumefantrine arm, 6 in the artesunate-amodiaquine arm, and 15 in the artesunate-sulfadoxine-pyrimethamine arm underwent direct skin feeding. The mean age of volunteers was comparable between groups (Table 1).

**TABLE 1:** Characteristics of the volunteers subjected to direct skin feeding assays.

Characteristics	Baseline (*n* = 21)	AL(*n* = 8)	ASAQ(*n* = 6)	ASSP(*n* = 15)
**Age (years)**				
Mean ± SD	7.7 ± 2.2	7.6 ± 1.4	6.8 ± 1.6	7.5 ± 1.1
Median *(min, max)*	7 *(6–14)*	7.5 *(6–10)*	6.0 *(6–10)*	8 *(6–10)*
**Sex (%)**				
Female	33.33	50.00	33.33	13.33
**Gametocyte density**				
Mean ± SD	21.8 ± 13.9	10.75 ± 5.5	22.83 ± 22.8	16.87 ± 17.2
Median *(min, max)*	23 *(8–53)*	8 *(8–23)*	15 *(8–68)*	8 *(8–68)*

AL, artemether-lumefantrine; ASAQ, artesunate-amodiaquine; ASSP, artesunate-sulfadoxine/pyrimethamine.

Overall we dissected 698, 253, 174, and 602 mosquitoes to measure oocyst positivity at baseline, post-artemether-lumefantrine, post-artesunate-amodiaquine, and post-artesunate-sulfadoxine-pyrimethamine treatment, respectively (Table 2).

**TABLE 2:** Mosquitoes dissected per treatment arm and days of feeding.

Study day	No. of mosquitoes used for direct skin feeding experiment			
	Baseline	AL	ASAQ	ASSP
Day 0	698	–	–	–
Day 3	–	87	32	140
Day 7	–	166	91	282
Day 14	–	–	51	99
Day 21	–	–	–	81
Total	698	253	174	602

AL, artemether-lumefantrine; ASAQ, artesunate-amodiaquine; ASSP, artesunate-sulfadoxine/pyrimethamine.

**FIGURE 2:**
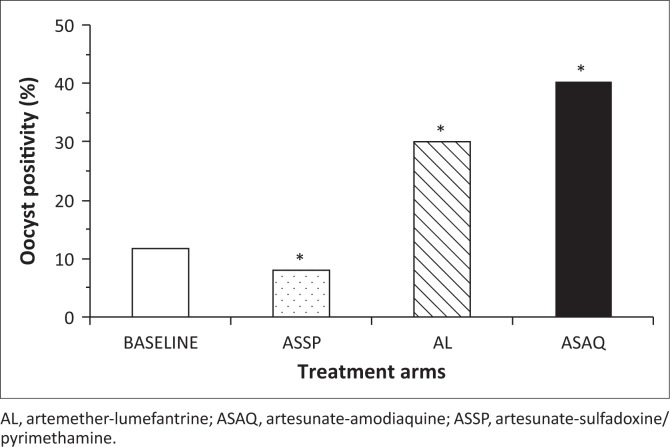
Oocyst positivity rate by treatment arm.

The oocyst positivity rate in the control group was 11.7% (82/698) (Figure 2). For volunteers treated with artemether-lumefantrine, the overall positivity rate was significantly higher than that of the control group (11.7%, *n* = 698 vs 30.0%, *n* = 253; *p* < 10^−4^) (Figure 2). Likewise the oocyst positivity rate following artesunate-amodiaquine treatment was significantly higher than that of the control group (11.7%, *n* = 698 vs 40.2% *n* = 174; *p* < 10^−4^) (Figure 2). In contrast, the oocyst positivity rate following artesunate-sulfadoxine-pyrimethamine treatment was significantly lower than that of the control group (11.7%, *n* = 698 vs 7.9%, *n* = 602; *p* = 0.03) (Figure 2).

## Discussion

The goal of this study was to determine the impact of different ACT regimens on *Plasmodium falciparum* gametocyte carriage, density and infectivity to *Anopheles gambiae s.l.* following artemether-lumefantrine, artesunate-amodiaquine and artesunate-sulfadoxine-pyrimethamine treatment. We measured *P. falciparum* gametocyte density of consenting volunteers suffering from acute uncomplicated malaria and gametocyte infectivity to *Anopheles* mosquitoes before and after ACT administration. From the direct skin feeding experiments of this study, we show that post-artemether-lumefantrine and post-artesunate-amodiaquine treatment gametocytes are more infectious to *Anopheles gambiae s.l.* than post-artesunate-sulfadoxine-pyrimethamine treatment gametocytes or non-drug treated controls. Since artemisinin derivatives, including artesunate and artemether, are rapidly metabolised *in vivo* into dihydroartemisinin, the observed differences in infectivity could be dependent on the partner drugs (i.e. lumefantrine, amodiaquine and sulfadoxine-pyrimethamine). However, one cannot rule out the effect of differences in artemisinin derivative pharmacokinetics and pharmacodynamics between regimens. Co-exposure to artemisinin derivative and the partner drugs albeit for a short period could impact gametocyte biology differently from any of the individual drugs. In addition, drug administration timing differs between artemether-lumefantrine and artesunate-amodiaquine or artesunate-sulfadoxine-pyrimethamine. While artemether-lumefantrine was given twice daily for 3 days, artesunate-amodiaquine and artesunate-sulfadoxine-pyrimethamine were administered once daily for 3 days. A number of studies have investigated the effect of antimalarial drugs on gametocyte density and infectivity.^19,31^ Beavogui et al. revealed that sulfadoxine-pyrimethamine considerably increased gametocyte carriage in the population but the infectivity of these circulating gametocytes was very low.^22^ Further investigation by Kone et al. showed that the low infectivity following sulfadoxine-pyrimethamine treatment was due to pyrimethamine, which is known to prevent male gametocyte exflagellation.^23^ In addition, 4-aminoquinoline chloroquine, which is chemically related to amodiaquine, has been shown to induce the production of fully infective gametocytes both *in vitro* and *in vivo*.^16^ These studies support the observed decrease of infectivity with artesunate-sulfadoxine-pyrimethamine treatment and the increase of infectivity with artesunate-amodiaquine seen in this study. Although gametocyte infectivity was different between the tested ACT regimens, the small number of carriers tested may be a limitation of this study. Additional infectivity studies with higher numbers of gametocyte carriers need to be conducted to further investigate these findings. In addition, the mechanisms involved in antimalarial drugs and gametocyte biology ought to be thoroughly studied.

To investigate gametocyte dynamics in the peripheral blood, gametocytes carriers were included in this study.^35^ Baseline gametocyte carriage was significantly higher in the artemether-lumefantrine arm compared to both artesunate-amodiaquine and artesunate-sulfadoxine-pyrimethamine. That observation may have occurred by chance as the study was a randomised controlled trial; therefore this could not be attributed to a selection bias.^35^ There was no difference in gametocyte density between the treatment arms at baseline. Gametocyte carriage significantly decreased in all treatment arms between Day 0 and Day 28. This result confirms numerous previous findings.^30^ However, while gametocyte prevalence in the artemether-lumefantrine arm decreased steadily from Day 0 to Day 28, there was a significant increase in the artesunate-amodiaquine and artesunate-sulfadoxine-pyrimethamine arms from baseline to Day 3 with a plateau until Day 7. This rise after ACT administration corroborates previous work^38,39^ and could be a consequence of the stress induced by the treatment.^26,40,41^ These data suggest that artemether-lumefantrine may affect gametocytogenesis differently from artesunate-amodiaquine or artesunate-sulfadoxine-pyrimethamine.^24,42,43^ Conversely, the dosing schedule of artemether-lumefantrine, which is taken every 8 h instead of once daily as for artesunate-amodiaquine and artesunate-sulfadoxine-pyrimethamine, could also play a role in the above observations.

We show that artesunate-amodiaquine and artemether-lumefantrine decreased gametocyte carriage in the treated population while both ACTs increased gametocyte infectivity to the mosquitoes. The effect of these ACTs in reducing gametocyte carriage can be a direct result of their active and fast killing action on asexual forms, leaving them no or little chance to differentiate into gametocytes.^30^ However, the few that manage to differentiate into gametocytes appear to be well fit for infecting the mosquitoes, hence their increased infectivity. Conversely, sulfadoxine-pyrimethamine sharply increases gametocytes but those gametocytes appear to be less fit for infecting the mosquitoes.^22, 23^ Pyrimethamine was also shown to hamper sporogonic development of *P. falciparum* in the Anopheles mosquitoes.^44^ These could explain the decrease in infectivity of post artesunate-sulfadoxine-pyrimethamine gametocytes.

Gametocytes were detected and quantified by light microscopy, which is an important limitation of this study. Thus, gametocyte carriage and gametocyte density are likely to be underestimated with our approach. The use of molecular tools^7,45,46,47^ and gametocyte quantification after purification^38^ could have yielded higher proportions of gametocyte carriers and gametocyte density.

We also cannot exclude the possible modulation of the mosquitoes’ gut microbiota by the ACTs used in this study. The *Anopheles* gut microbiota is known to influence the sporogonic cycle^15^ and some drugs, such as antibiotics, have been shown to change the composition of mosquitoes’ gut resident microbes.^48^

### Conclusion

Antimalarial drugs influence gametocytogenesis and their impact on gametocyte density and viability are likely to differ from one combination therapy regimen to another. Evaluation of transmission potential in malaria-endemic areas requires more studies assessing the influence of current antimalarial treatment on gametocyte development and clearance *in vivo,* and the infectivity of post-treatment gametocytes. Understanding the potential impact of antimalarial drugs on the spread of resistant strains and malaria transmission will require a fine assessment of their effects on gametocyte biology and the mechanisms involved.
